# Artificial intelligence in antiphospholipid syndrome: toward individualized risk prediction

**DOI:** 10.1016/j.rpth.2025.103213

**Published:** 2025-10-13

**Authors:** Luis Enrique Gutierrez-Rosas, Froylan D. Martínez-Sánchez

**Affiliations:** 1 Facultad de Ciencias de la Salud, Universidad Anáhuac México Norte, Lomas Anáhuac, Huixquilucan, Estado de Mexico, Mexico; 2 Department of Internal Medicine, Hospital General “Dr. Manuel Gea González,” Mexico City, Mexico; 3 Facultad de Medicina, Universidad Nacional Autonoma de Mexico, Copilco Universidad, Coyoacán, Ciudad de México, Mexico

Antiphospholipid antibody syndrome (APS) is the most common form of acquired thrombophilia, characterized by arterial or venous thrombosis, pregnancy complications, and the persistent presence of at least 1 type of antiphospholipid antibody [1,2].

In 2023, the American College of Rheumatology (ACR) and the European Alliance of Associations for Rheumatology (EULAR) proposed updated classification criteria that provide a more structured framework for identifying patients with APS [2]. These criteria require the presence of at least 1 clinical and 1 laboratory domain within a 3-year window. The clinical domains encompass macrovascular and microvascular thrombosis, obstetric morbidity, cardiac valve disease, and hematologic abnormalities. Laboratory domains include the presence of lupus anticoagulant and moderate to high titers of anticardiolipin or anti-β2 glycoprotein I antibodies. For classification, patients must reach a minimum threshold of 3 points in both the clinical and laboratory domains [2].

Despite advances in anticoagulation, patients with thrombotic APS continue to face a high risk of recurrent events [1,3]. Recurrent events in APS have been associated with a range of clinical and biological factors. These include the breadth and persistence of the antiphospholipid antibody profile, the presence of antiprothrombin antibodies, variations in anticoagulant regimen and intensity, concomitant comorbidities such as renal disease, and patient-related characteristics, notably age [4]. Yet, recurrence remains frequent even when these risk factors are taken into account. This reflects a significant unmet need in APS: the inability of traditional prediction models to fully capture the multifactorial and nonlinear nature of the disease [5,6]. Consequently, there is an urgent need for novel approaches that can integrate diverse clinical and laboratory variables to more accurately predict recurrence and guide individualized treatment strategies.

The study by Marco-Rico et al. [6] addressed this unmet need by applying machine learning (ML) to predict thrombotic recurrence in patients with thrombotic APS. In a cohort of 72 anticoagulated patients followed at a single center in Spain, the authors compared several algorithms (support vector machine, random forest, k-nearest neighbor, and extreme gradient boosting) to assess their ability to discriminate recurrence risk (Figure). Rather than relying on a limited set of linear predictors, the models incorporated a broad spectrum of demographic, clinical, and laboratory variables, including age, sex, cardiovascular risk factors, autoimmune comorbidities, renal function, antiphospholipid antibody profile, and anticoagulant regimen.

**Figure:**
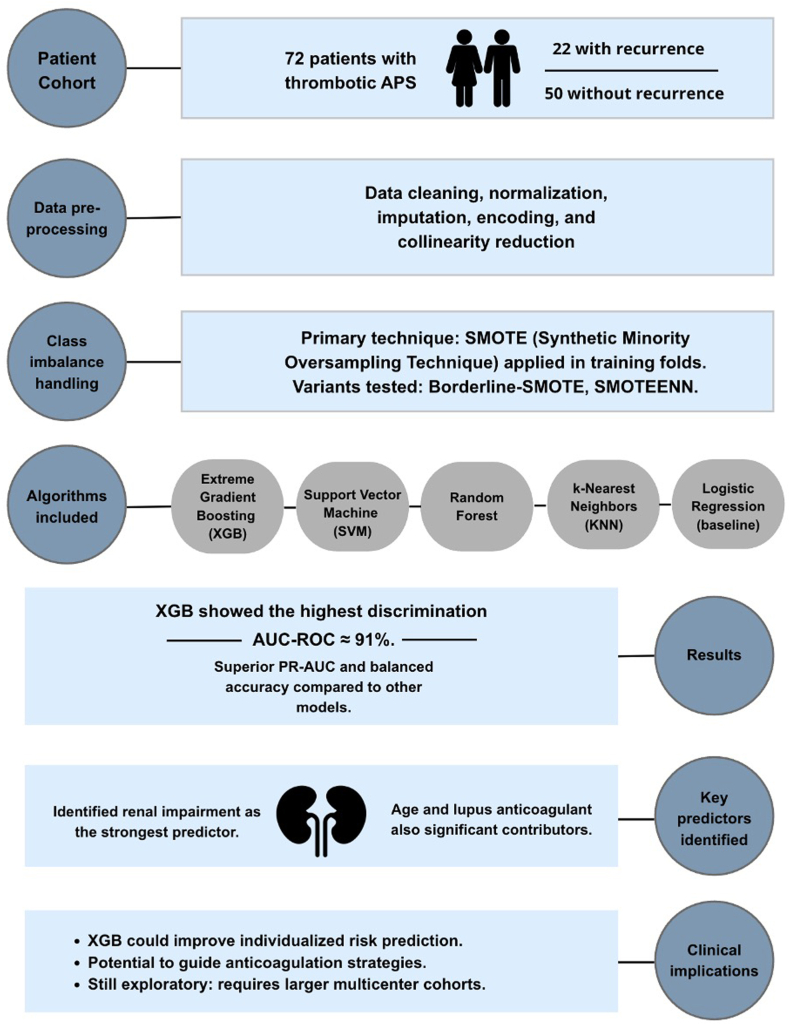
Workflow of predictive modeling for thrombotic recurrence in antiphospholipid antibody syndrome (APS). Pipeline from patient selection (72 thrombotic APS patients: 22 recurrence/50 nonrecurrence) to data preprocessing (cleaning, normalization, imputation, encoding, and collinearity reduction), class imbalance handling (synthetic minority oversampling technique [SMOTE]), machine learning model comparison (extreme gradient boosting [XGB], support vector machine [SVM], random forest, k-nearest neighbor [KNN], and logistic regression; nested cross validation [CV], 200 runs), model performance (XGB area under the receiver operating characteristic curve [AUC-ROC] ≈ 91%), interpretability (top predictors: renal impairment, age, and lupus anticoagulant), and clinical implications (potential for individualized risk prediction; requires external validation). PR-AUC, precision-recall area under the curve; SMOTEENN, synthetic minority over-sampling and edited nearest neighbors.

The group of Marco-Rico et al. [6] implemented rigorous preprocessing strategies to address missing data, collinearity, and class imbalance using techniques such as synthetic minority oversampling technique, and evaluated model performance through repeated nested cross-validation. Among the various approaches, extreme gradient boosting consistently yielded the best results, with an area under the receiver operating characteristic curve of approximately 0.91. Importantly, the model highlighted renal impairment, age, and lupus anticoagulant positivity as the most influential predictors of recurrence, findings that are clinically plausible and resonate with previous evidence [1,7].

However, these promising results should be interpreted with caution. The relatively small sample size, single-center design, and limited number of clinical variables inevitably limit the generalizability of the findings. Moreover, important markers, such as the international normalized ratio, did not emerge as predictors, underscoring the need to incorporate broader datasets and complementary biomarkers in future analyses. Ultimately, this work should be regarded as a proof-of-concept that requires external validation in larger and more diverse cohorts before it can be translated into clinical practice.

Artificial intelligence (AI) is increasingly being applied in medicine to support diagnostic and therapeutic decision-making [8,9]. Rather than aiming for autonomy, most current applications are designed to function as clinical aids, supporting physicians in integrating complex data into actionable insights. These systems are generally classified under the paradigm of “weak AI,” as they are restricted to specific, well-defined tasks, such as diagnostic support, image recognition, or risk stratification, without intentionality or consciousness [9]. Within this framework, ML emerges as a particularly powerful approach. By leveraging algorithms capable of identifying nonlinear interactions and hidden patterns in high-dimensional data, ML provides the opportunity to address focused clinical challenges that often elude conventional statistical models. Importantly, the goal is not to replace clinical judgment, but rather to create reliable, transparent, and clinically useful tools that complement physicians’ expertise, optimize patient care, and ultimately improve outcomes [[8], [9], [10]].

In rheumatology and thrombosis, the integration of digital health technologies has markedly expanded the ability to monitor patients beyond the clinic [11,12]. Tools such as telemedicine platforms, wearable sensors, mobile health applications, and continuous monitoring systems enable the passive and automated collection of longitudinal, real-time data with a level of granularity that far exceeds conventional clinical encounters. These innovations not only enhance patient engagement and adherence but also generate rich datasets that are ideally suited for advanced computational approaches. Recent work highlights that, when coupled with AI and ML, these technologies can be leveraged to detect early indicators of disease flares, model trajectories of organ damage, and predict therapeutic responses with greater precision than traditional clinical predictors [10,11]. Importantly, their potential extends beyond rheumatology, as similar applications are emerging in thrombosis and hemostasis, underscoring the broader relevance of AI across specialties [10].

For patients with APS, the clinical burden of recurrent thrombosis remains considerable [1,7]. Several cohort and interventional studies have shown that thrombotic recurrence in APS remains substantial despite anticoagulation, with rates varying across populations and treatment regimens. Outcomes are influenced by the breadth of the antiphospholipid antibody profile, the intensity and type of anticoagulant therapy, and concomitant comorbidities, underscoring the need for more effective and individualized strategies [4,13]. Incorporating ML into risk prediction models could meaningfully alter this landscape, allowing for more accurate stratification of recurrence risk (and other APS-associated complications), optimization of anticoagulant therapy, prevention of complications, and dynamic adaptation as new longitudinal data accumulate [14,15]. Such approaches could ultimately complement existing clinical guidelines by providing individualized decision support tailored to each patient’s risk profile.

Overall, the study by Marco-Rico et al. [6] exemplifies how AI can advance precision medicine in APS. By moving beyond linear models and static predictors, ML approaches have the potential to transform risk stratification, guide therapeutic strategies, and improve long-term outcomes in this complex and high-risk population [16,17]. While challenges remain regarding external validation, data sharing, and clinical integration, the trajectory is clear: computational tools will increasingly shape the way we approach APS and thrombosis. The challenge for the coming years will be to balance innovation with rigorous clinical validation, ensuring that these technologies translate into safer, more effective, and more equitable care for patients worldwide.
